# Supplementary material for: Mid infrared gas spectroscopy using efficient fiber laser driven photonic chip-based supercontinuum
Source: Nat Commun. 2019 Apr 4;10:1553. doi: 10.1038/s41467-019-09590-3 (PMC6449389; doi:10.1038/s41467-019-09590-3)
Supplement: Supplementary file 1 — Supplementary Information [file 41467_2019_9590_MOESM1_ESM.pdf]

Supplementary Information:

Mid infrared gas spectroscopy using efficient fiber laser driven photonic chip-based supercontinuum

Grassani et al.

SUPPLEMENTARY NOTE 1: INFLUENCE OF SELF-STEEPENING AND GROUP VELOCITY DISPERSION IN DISPERSIVE WAVE GENERATION DYNAMICS

In this note, we provide a more detailed explanation of the role of self-steepening in estimating the spectral position of the mid-IR DW. Also, we comment about the role of $|\beta_2|$ in affecting the soliton compression point and the efficiency of the DW generation process. At this purpose, we numerically simulate the pulse evolution in the investigated waveguides by considering the same coupled pump power of about 12 mW. We chose such pump power to keep the soliton number (*N*) below 10 for all the investigated waveguides, and compare their dynamics within the same regime, namely soliton induce DW generation.

Supplementary Figs.1 (a)-(d) clearly show that the discrepancy in the position of DW generation observed between Fig.3a and b in the main text mainly come from the larger blue shift of the central soliton due to the stronger recoil, not included in the phase matching condition of Equation 1 in the main text, underwent in smaller waveguides, and given by the larger soliton number and efficiency in power transfer to the DW. In fact, comparing the experimental data with the spectrum obtained by numerically solving the Nonlinear Schrödinger Equation (NLSE), we see how the predicted DW well reproduces the position of the experimental one for all the waveguides. Here, slight differences can be due to variation of the actual waveguide size with respect to the nominal one, due to the tolerance on the lithography (about 25 nm). The wavelength shift due to the soliton recoil can be observed in the spectra reported in Supplementary Figs.1 (e)-(h) too. Also, this effect can be noticed in the temporal domain shown in Supplementary Figs.1 (i)-(n). In fact, after a first slowdown of the soliton pulse due to an increase of its group index due to self-steepening, the pulse accelerates reducing its delay in the proximity of the compression point. This is a signature of recoil, as a spectral blue-shift accelerates a pulse placed in the anomalous dispersion region. Consistently with the above mentioned arguments, this bend in the pulse trajectory is more pronounced in the smaller waveguides.

Also, the spectral evolution in Supplementary Figs.1 (e)-(h) shows that, although soliton fission happens at a slightly longer distance, there are more compression points in smaller waveguides, increasing the overall power transfer to the DW. Both of these behaviors can be explained with the decreasing value of $|\beta_2|$ when decreasing the width of the waveguides. In fact, both the soliton fission distance (l_c) and soliton number (N) are inversely proportional to $|\beta_2|$: $l_c \propto \left(|\beta_2|P\right)^{-1/2}$ and $N \propto \left(P/|\beta_2|\right)^{-1/2}$. This is in agreement with the experimental data of Fig.4 in the main text, where the DW generation efficiency is already around 30 % for 12 mW of coupled pump power in the 1000 nm and 1050 nm width waveguides. Further increasing the pump power will lead to a noise-seeded dynamics for DW generation in smaller waveguides, but to multiple compression points in larger waveguides, which can eventually reinforce longer DWs.

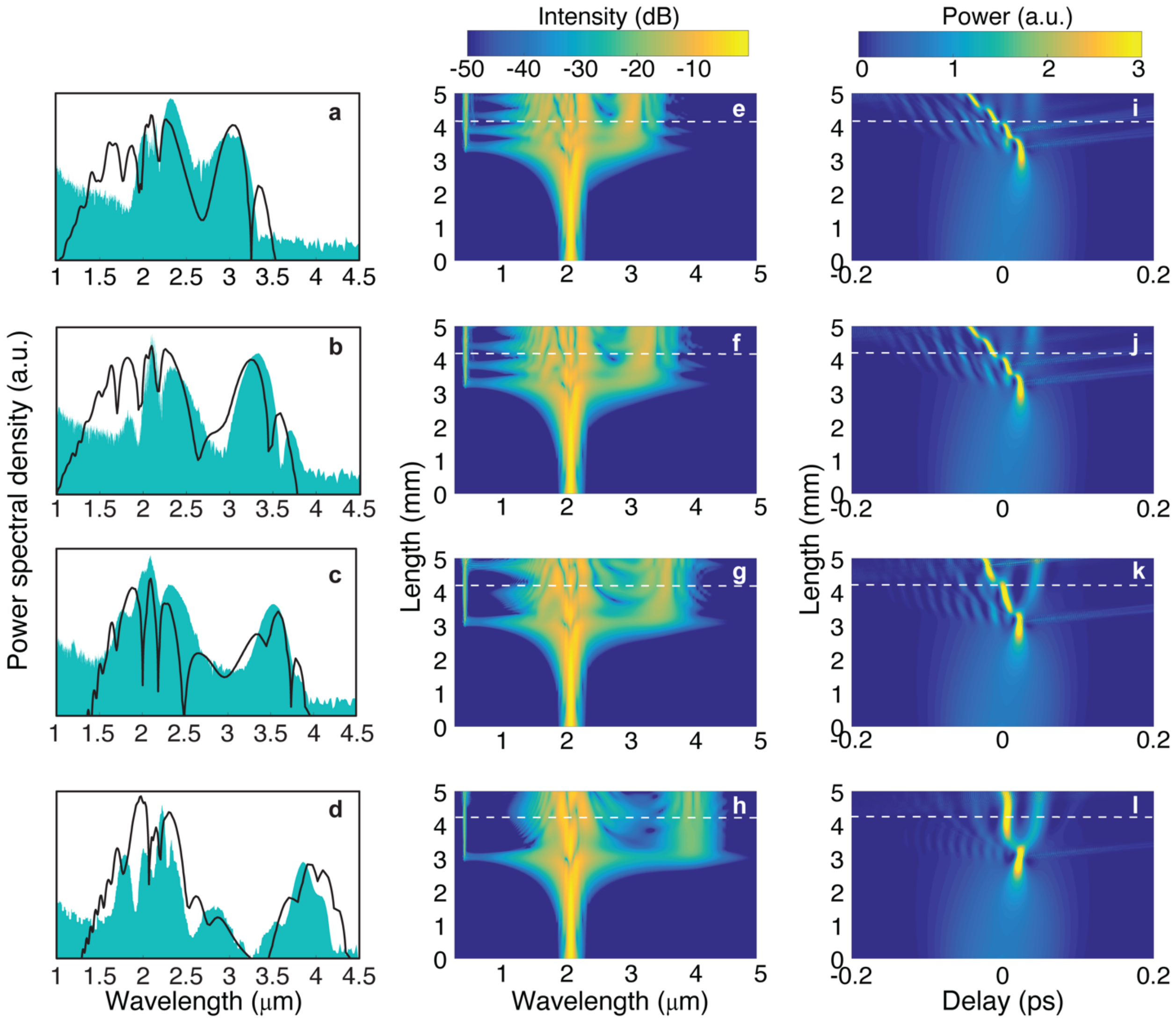

SUPPLEMENTARY FIGURE 1. SOLITON DYNAMICS COMPARISON. (a)-(d) Spectra measured at the output of the waveguides (filled green areas) superimposed with the numerical simulation after 4.2 mm of pulse propagation (black lines). Numerical pulse propagation evolution along the waveguide as a function of wavelength (e-h) and time (i-l). The dotted white line corresponds to the effective length considered for propagation (4.2 mm). The four waveguides, from top row to bottom row, have dimensions: 2090 x 1000 nm^2, 2120 x 1050 nm^2, 2150 x 1100 nm^2, 2190 x 1175 nm^2.
